# Reliability and validity of the Chinese version of the LMC Skills, Confidence & Preparedness Index (SCPI) in patients with type 2 diabetes

**DOI:** 10.1186/s12955-020-01664-x

**Published:** 2021-01-20

**Authors:** Ximin Wang, Weibo Lyu, Ronnie Aronson, Aihua Li, Gendi Lu, Weijin Xu, Yang Cao, Ying Yu, Liting Wang, Huiting Lin

**Affiliations:** 1 School of Nursing, Shanghai University of Traditional Chinese Medicine, 1200 Cailun Road, Zhangjiang Hi-Tech Park, Pudong New Area, Shanghai, 201203 China; 2 LMC Diabetes & Endocrinology, Toronto, ON Canada; 3 Shuguang Hospital, Shanghai University of Traditional Chinese Medicine, Shanghai, China; 4 Clinical Epidemiology and Biostatistics, School of Medical Sciences, Örebro University, Örebro, Sweden

**Keywords:** Diabetes, Self-management, Assessment tool, SCPI, Translation, Cross-cultural adaption, Validity, Reliability

## Abstract

**Background:**

A variety of diabetes self-management instruments have been developed but few of them consist of the preparedness for diabetes self-management behavior. The novel psychometric evaluation tool “the LMC Skills, Confidence & Preparedness Index (SCPI)” measures three key aspects of a patient’s diabetes self-management: knowledge of the skill, confidence in being able to perform skill and preparedness to implement the skill. The objective of this study was to translate, adapt and validate the SCPI for use in Chinese adult patients with type 2 diabetes.

**Methods:**

This study followed the guideline recommended by the American Academy of Orthopaedic Surgeons Evidence Based Medicine Committee (AAOS) to indigenize the scale. Forward and back translation, and cross-cultural language debugging were completed according to the recommended steps. A convenience sample of Chinese patients with type 2 diabetes (n = 375) were recruited from a university-affiliated hospital in Shanghai. The validity (criterion, discriminant validity, and construct validity), reliability (internal consistency and test–retest reliability) and the interpretability of the instrument were examined. The content validity was calculated by experts’ evaluation.

**Results:**

The Chinese version of SCPI (C-SCPI) has good internal consistency with a Cronbach’s alpha of 0.92. The ceiling effects of the preparedness subscales is 21%. The criterion validity of three dimensions of C-SCPI was established with significantly moderate correlations between the DKT, DES-SF and SDSCA (*p* < 0.05). The S-CVI of the whole scale was 0.83. Except for entry 21, the I-CVI values of all entries were greater than 0.78. The C-SCPI has also shown good discriminative validity with statistically significant differences between the patients with good and poor glycemic control. Confirmatory factor analysis showed that modified results indicate that the fitting degree of the model is good, χ^2^/df = 2.775, RMSEA = 0.069, CFI = 0.903, GFI = 0.873, TLI = 0.889, IFI = 0.904. The test–retest reliability coefficient was 0.61 (*p* < 0.01).

**Conclusion:**

We established a Chinese version of SCPI through translation and cross-cultural adaptation. The C-SCPI is reliable and valid for assessment of the level of self-management in Chinese patients with type 2 diabetes.

## Background

Diabetes mellitus is a non-communicable disease and is becoming epidemic worldwide. The latest global diabetes atlas (9th Edition) released by the International Diabetes Federation (IDF) showed that the prevalence of diabetes is increasing rapidly, with an average global growth rate of 51% and the number of diabetics in China ranks first in the world, with a total population of about 116.4 million in 2019 [1], which forms a heavy burden for families, society and the whole country in China [2]. Many experts unanimously recommend improving the self-management level of diabetic patients as the main way to prevent and treat diabetes [3–5]. In 1996, IDF proposed that self-management for diabetes should include diet, exercise, medication, diabetes health education and self-glycemic monitoring [6]. The current status of self-management of diabetes is not satisfactory in China [7–9].

Accurate assessment of the patient’s current self-management level is an indispensable part of health education. Chinese consensus on self-administered prescriptions for type 2 diabetes also describes the importance of a comprehensive, systematic assessment of patients before developing a personalized self-management program [10]. The scientific and standardized assessment tool is the key to assessing the self-management level of patients. Nowadays, diabetes self-management education consists of three parts: the conveying of knowledge, the establishment of health beliefs, and the guidance of behavior change. Particularly, behavior change is considered to be a sign of success in measuring the impact of diabetes education programs [11]. Therefore, diabetes-related self-management assessment tools generally focus on knowledge, psychology and behavioral changes.

The current assessment tools have some limitations. Firstly, many tools are unidimensional. Some focus on knowledge [12–14], some on attitude and belief [15–17] and some on practice [18–20]. The Diabetes Care Profile(DCP) [21], a comprehensive assessment tool has a solid theoretical basis and a rich measurement dimension, but the too many items in this scale impede its practical application. Secondly, the reliability and validity of some scales were only verified at the time of the scale development, limiting their applications in different contexts. Some scales showed bias in reliability and validity [19]. Thirdly, the scales about practice focus most on the pre-existing behaviors [18, 20, 22], while less attention is paid to the level of preparation for further behavioral changes. For example, in the Diabetes Self-management Knowledge, Attitude, and Behavior Assessment Scale (DSKAB) [22], which was developed by Chinese scholars, patients are asked to recall their behaviors within half a year, a long time span which may result in a memory bias for patients. Researchers [23]have pointed out that accurately assessing patients’ ability and readiness before starting self-management behavior is a prerequisite for developing and implementing any patient-centered approach to self-management.

We chose the LMC Skills, Confidence & Preparedness Index (SCPI) [24, 25] for translation, adaption and validation in Chinese population, because it is the first “all in one” scale to evaluate three key aspects of diabetes self-management simultaneously. Knowledge of the skills is based on the content of the seven self-care activities of the AADE and the core content of diabetes self-management and Canadian Clinical Practice Guidelines (CPG) [26], including healthy diets, medications, activities, blood glucose monitoring, problem solving, and risk reduction and healthy coping. Confidence in being able to perform the skills is based on the self-efficacy theory (SET) [27]. Self-efficacy is the subjective self-confidence that an individual believes he or she can perform certain behaviors and achieve the desired results. Preparedness to implement the skills is based on the preparation phase originally derived from Transtheoretical Model of Health Behavioral Change which means individuals will take action to change behavior within one month [28]. It measures the motivation for behavioral change and the degree to which patients will make changes next month. This dimension involves diet, exercise, stress relief, prevention of hypoglycemia, and insulin use when necessary. This scale had been developed in LMC diabetes and endocrine clinics in Ontario, Canada and validated in 2 more independent cohorts. Its clinical responsiveness to a diabetes education program intervention was investigated in 51 patients. They were assessed by SCPI before the implementation of health education program, so that educators can quickly identify the existing difficulties of diabetes patients in knowledge, skills, confidence and behavior preparation. Especially in the aspect of behavior, it focuses on the motivation of behavior change, through the evaluation of “behavior preparation” stage of diabetic patients, we can understand the needs of patients or provide basis for health educators to take corresponding strategies, so that medical staff can know “what to teach first”. On the basis of education project, more personalized education is provided for patients. After 3 months, patients’ glycosylated hemoglobin level have been significantly improved (9.3 ± 1.0% vs 8.2 ± 0.9%, *p* < 0.001) [25].

SCPI has been well validated in Canada, which has an important role in evaluating the status quo of self-management and behavior preparation of diabetic patients, and it can play a certain role in prompting health educators to carry out education for patients. The application in China is worth further exploration. Thus, the objective of this study was to adapt the “The LMC Skills, Confidence & Preparedness Index” (SCPI) into Chinese with type 2 diabetes and validate its psychometric properties.

## Methods

The research was approved by the developer of the questionnaire. This is a two-phase study. In phase one, we performed a trans-language adaption of SCPI. In phase two, the psychometric properties of the Chinese version of SCPI were validated.

### Phase one: trans-language adaption of SCPI

The cross-cultural adaption process of the scale is a process of examining the equivalence between the indigenized scale and the original scale. In this phase we followed a systematic process from the guideline recommended by American Academy of Orthopaedic Surgeons Evidence Based Medicine Committee (AAOS) [29] which included forward-translation, synthesis of the translations, back-translation, expert committee and testing of the translated version.

1. *Forward translation* The English version of each of the SCPI questions was translated into Chinese by two independent translators who are native speakers of Chinese and they used plain language to express original meaning to the greatest extent. They generated translation versions T1 and T2.
2. *Synthesis of the translations* A nursing expert with high English proficiency and overseas experience reviewed two translation versions T1 and T2 and compared them with the original scale to highlight any ambiguous wordings. The research team conducted a collective discussion on the original English, two translations, and the expert’s revision opinions, and discussed the uncertainties and disputes. After reaching an agreement, it was moved to form a Chinese translation version T3.
3. *Back translation* The reconciled version was then back-translated by three individuals: a nursing graduate student with overseas study experience, a nursing expert living in the United States and a Chinese-American who has no medical background. They generated translation versions T3-1, T3-2 and T3-3. None of the three had read the original English scale. Then, representative authors of the source scale and the nursing experts who are familiar with the subject matter compared the accuracy of the three back-translation versions and discussed any uncertainties with the members of the research group. The final version of T4 was formed according to the opinions of language coordinators.
4. *Expert committee* We invited six experts who are proficiently bilingual to form a panel of experts, including two endocrinologists, three clinical nursing experts engaging in endocrine practice, and a chronic disease management expert. The experts evaluated the Chinese translation and the back translation scale using the 5-point Likert rating according to the English original version. The five options are: fully consistent, very consistent, basically consistent, less consistent, inconsistent, corresponding to five to one point respectively. Experts provided comments and suggestions for ambiguous items. After being reviewed, the Chinese translation scale was revised based on the opinions and suggestions from the expert panel.
5. *Testing of the Chinese version* We invited 15 patients with type 2 diabetes to complete the scale, and then used an interview process to assess the patients’ understanding of the scale descriptions, entries, and responses to know if the text of the scale was easy to understand, and whether the entries were ambiguous or unclear. All respondents were asked to answer a series of open-ended questions. (1) Do you understand this item? (2) If there are any difficulties, how would you reword it? (3) Do you think that the items in the scale are irrelevant or did the description make you feel uncomfortable? (4) If there are any difficulties, can you tell me which items are in appropriate and why? Can you provide an appropriate expression? The Chinese version of the scale was finalized based on the further revision following the interview process. The general information of the experts and the whole process of trans-language adaption of SCPI were shown in the Additional file 1.

### Phase two: assessment of the reliability and validity evaluation of SCPI

In this phase, the psychometric properties of the SCPI were tested. The target population was patients with diabetes. A convenience sampling was performed in a university-affiliated hospital in Shanghai, China between June 2018 and December 2019. Eligible patients are those who are 18 years or older and in line with WHO diagnostic criteria for diabetes (1999) [30], type 2 with a course over 6 months. Those with serious complications such as cardiac function (NYHA3 or above), renal function (CKD4 or above), cardiovascular and cerebrovascular diseases or organ function damage were excluded.

Per rule of thumb, it is highly recommended to use at least 10 subjects per item of the instrument for general psychometric approaches. If there is a plan to use confirmatory factor analysis to test the factor structure of the instrument, the recommendation per rule of thumb is approximately 300–500 subjects per item of the instrument [31]. SCPI has 23 items in total, the sample size of this study needs at least 230 subjects. In addition, this study needs confirmatory factor analysis, and the sample needs at least 300 subjects. Considering an invalid response rate of 20%, the sample size was finally determined to be at least 360.

### Instrument

#### The LMC Skills, Confidence & Preparedness Index (SCPI)

LMC is a multidisciplinary, regional community-based sites providing comprehensive specialist-level care for patients with diabetes in Canada. SCPI [25] includes 23 items in total, which measure three key aspects of a patient’s diabetes self-management: knowledge of the skill (9 items), confidence in being able to perform the skill (7 items), and preparedness to implement the skill (7 items). The responses to each item were recorded on a seven-point Likert scale (range: 1 = strongly disagree, 7 = strongly agree).

#### Diabetes Knowledge Test (DKT)

DKT [12] was developed by members of Michigan diabetes research and training center in the United States. DKT has 23 items, including knowledge on diet, blood glucose monitoring, exercise, prevention and treatment of complications, insulin and other knowledge. The test is divided into two parts. The first part includes 14 items, which are applicable to adult patients with type 1 and type 2 diabetes. The other nine items constitute the insulin use subscale. The higher the score, the better the patient’s disease knowledge. Chen [32] translated and used it among Chinese people. DKT is used to validate the criterion validity of the knowledge and skills dimension of SCPI [24].

#### Diabetes Empowerment Scale (DES-SF)

DES [17] was developed by Anderson in 1991 to measure the social and psychological self-efficacy of diabetic patients. In order to make the evaluation more convenient, Anderson reduced the scale to a simplified 8-item version named DES-SF. Hu [33] introduced and translated DES-SF in Chinese. A higher score indicates a better patient’s self-efficacy. DES-SF is used to validate the criterion validity of the confidence dimension of SCPI [24].

#### The Summary of Diabetes Self-Care Activities Measure (SDSCA)

SDSCA [18] is widely used in the world and has shown good reliability and validity in China. In Hua’s study, Cronbach’s α of the Chinese version of SDSCA was 0.918 [34]. The scale consists of 11 items, including general and special activities in diet, exercise, blood glucose monitoring, foot care, and medication. A higher score indicates a better self-management behavior. SDSCA is used to validate the criterion validity of the preparedness dimension of SCPI. The preparedness part of SCPI reflects the degree of behavioral preparation of patients in the next month, and the SDSCA reflects the level of diabetic self-management through the frequency of self-care activities of patients in the first 7 days before reporting. Therefore, one month after the completion of the SCPI, some patients were investigated again with the SDSCA to verify whether the patient’s behavioral preparation was related to the subsequent self-management activities.

Sociodemographic data such as gender, age, education level, monthly income and whether there is health education for diabetes were self-reported by the participants. Clinical data of the participants such as HbA1c were collected from the hospital’s electronic medical records. We explained the purpose and the contents that need to be cooperated clearly to the patients, and promise to protect their privacy before the investigation. The patients check the corresponding options in accordance with their own daily self-management of diabetes on the paper by themselves. We would guide patients to fill in the questionnaire if they were unable to read.

#### Statistical analysis

The general characteristics of the subjects were presented using mean and standard deviation for continuous variables, and frequency and percentage for category variables. The Kolmogorov–Smirnov Test was used to examine the normality of data distribution. To assess models’ goodness of fit, confirmatory factor analysis (CFA) was performed with the following indices: goodness-of-fit index (GFI), comparative fit index (CFI), incremental fit index (IFI), Tucker-Lewis index (TLI) and root mean square error of approximation (RMSEA). An acceptable model should have a χ^2^/df < 3, RMSEA < 0.08, and GFI, CFI, IFI and TLI > 0.9 [35]. The validity tests also include content validity, criterion validity, and discriminative validity. The content validity of the Chinese version of the SCPI was evaluated using the content validity index (CVI), which includes I-CVI (content validity of individual items, i.e. proportion of experts giving a rating of either 3 or 4) and S-CVI (content validity of the overall scale, i.e. proportion of items in a scale that achieves a relevance rating of 3 or 4 by all the experts) [36]. Correlational analysis of the Chinese version of SCPI and DKT, DES-SF & SDSCA was applied to examine the criterion validity of the SCPI. We used Pearson’s correlation analysis for normally distributed data, and Spearman’s non-parametric correlation for data not normally distributed. Discriminative validity of the SCPI was tested using a nonparametric test (Mann–Whitney U test) to compare the SCPI scores between patients with satisfactory blood glucose control (HbA1c ≤ 7) and patients with poor blood glucose control (HbA1c > 7). Cronbach’s α was used to measure the internal consistency reliability. The SCPI was repeated after 2 weeks to evaluate its test–retest reliability by 23 participants. Distributional methods look at the statistical distribution of the instruments values. The standard error of measurement (SEM) and one-half of standard deviation (SD) of the measure of interest are most widely accepted to represent minimal clinically important difference (MCID) values [37]. SEM was calculated using SD at baseline of SCPI score × (1-reliability of the validated Chinese version of SCPI)^1/2^. Statistical tests were performed using the SPSS 24.0 and Amos 24.0 for Windows (IBM). A two-sided *p* value of < 0.05 was considered statistical significance.

## Results

### Phase one: trans-language validation of SCPI

The translation process led to a Chinese version of the SCPI that was linguistically validated and conceptually equivalent to the original version. In the process of synthesis, one of the two translations was selected for 8 (34.8%) items (3 from the first and 5 from the second translator, respectively), and a combination of translations from the both translators was use for 15 (65.2%) items. The result of back translation was similar to the original English version.

Eight changes were made after the expert committee. In the expert ratings, the average score of all items in the translation process is larger than 3 points, and 74% of the items have score larger than 4 points, which is very consistent with the original text. In the back-translation section, all items have points above 3, and 61% above 4 points. In addition to language modification, the change of “sickness” to “physical discomfort”in item 6 could make patients pay more attention to the subtle changes of their bodies. In item 21, two experts pointed out that patients should not be encouraged to adjust their insulin doses by themselves because insulin dosage adjustment should be considered according to the patient’s condition and the type of insulin used. In light of the clinical situation that patients should adjust the dosage of insulin under the guidance of doctors in China, the panel decided to change “I will start adjusting my insulin doses on my own” to “I will start adjusting my insulin doses on my own as recommended by my doctor.”

In the test for the final translated version, after 15 patients with type 2 diabetes completed the questionnaire, we conducted interviews with them. Nine males and six females, aged from 28 to 70 years, had diabetes for 10.37 ± 8.57 years. Two individuals had a primary school education, nine had high school education, two completed college diploma, and two had a bachelor’s degree.

Six additional modifications (see the Additional file 1) were made after interviews with the patients. Three patients did not understand the meaning of carbohydrates. Following the principle of experimental equivalence [29], we explained the meaning of carbohydrates in detail and interpreted carbohydrates as foods containing starch/sugar. Some were not sure about the scope of self-management of diabetes, therefore we elaborated the scope of self-management of diabetes, such as diet, medication, exercise, blood sugar monitoring. And seven patients were doubtful about some expressions like “blood sugar pattern”, “keep my diabetes on track”, and “stress management”. We changed the “blood sugar pattern” to “change of blood sugar” and explained it (such as the cause of hyperglycemia or hypoglycemia). Patients had also expressed that “keep my diabetes on track” was not in line with Chinese language habits, so we changed it to “control blood sugar within the target range”. We interpreted “stress management” as “a way to relieve stress”.

### Phase two: assessment of the internal consistency reliability and construct validity of the SCPI

Characteristics of the convenience sample at baseline are reported in Table 1. A total of 375 participants completed the SCPI. All patients were type 2 diabetes patients with a mean HbA1c of 8.5 ± 1.9% (excluding 75 without HbA1c measurement), and 218 of them were insulin users. The mean age is 57.2 ± 12.7 years, and mean duration of diabetes is 11.5 ± 8.0 years. Most patients (65.6%) have received health education on diabetes.

**Table 1:** Characteristics of participants at baseline (N = 375)

	T2Di (N = 218)	T2D (N = 157)	Total (N = 375)
HbA_1C_ (T2Di N = 169; T2D N = 131)	8.8 ± 1.9	8.2 ± 1.9	8.5 ± 1.9
Age (N = 375)	58 ± 13	56 ± 12	57.2 ± 12.7
Sex (N = 375)			
Male (%)	141 (64.7%)	99 (63.1%)	240 (64.0%)
Female (%)	77 (35.3%)	58 (36.9%)	135 (36.0%)
Diabetes duration (years) (N = 375)	13.5 ± 8.1	8.7 ± 6.8	11.5 ± 8.0
Education (N = 375)			
University degree or above	60 (27.5%)	50 (31.8%)	110 (29.3%)
High school	64 (29.4%)	34 (21.7%)	98 (26.1%)
Junior high school or below	94 (43.1%)	73 (46.5%)	167 (44.5%)
Income (per month) (N = 375)			
CNY 0–3000	29 (13.2%)	28 (17.8%)	57 (15.2%)
CNY 3000–6000	59 (27.1%)	45 (28.7%)	104 (27.7%)
CNY 6000–8000	33 (15.1%)	22 (14.0%)	55 (14.7%)
CNY > 8000	97 (44.5%)	62 (39.5%)	159 (42.4%)
Diabetes education (N = 375)			
Yes	143 (65.6%)	94 (59.9%)	237 (63.2%)
No	75 (34.1%)	63 (40.1%)	138 (36.8%)

T2Di means Type 2 diabetic patients using insulin; T2D means Type 2 diabetic patients without insulin

### Factor structure of the Chinese version of SCPI

**Fig. 1:**
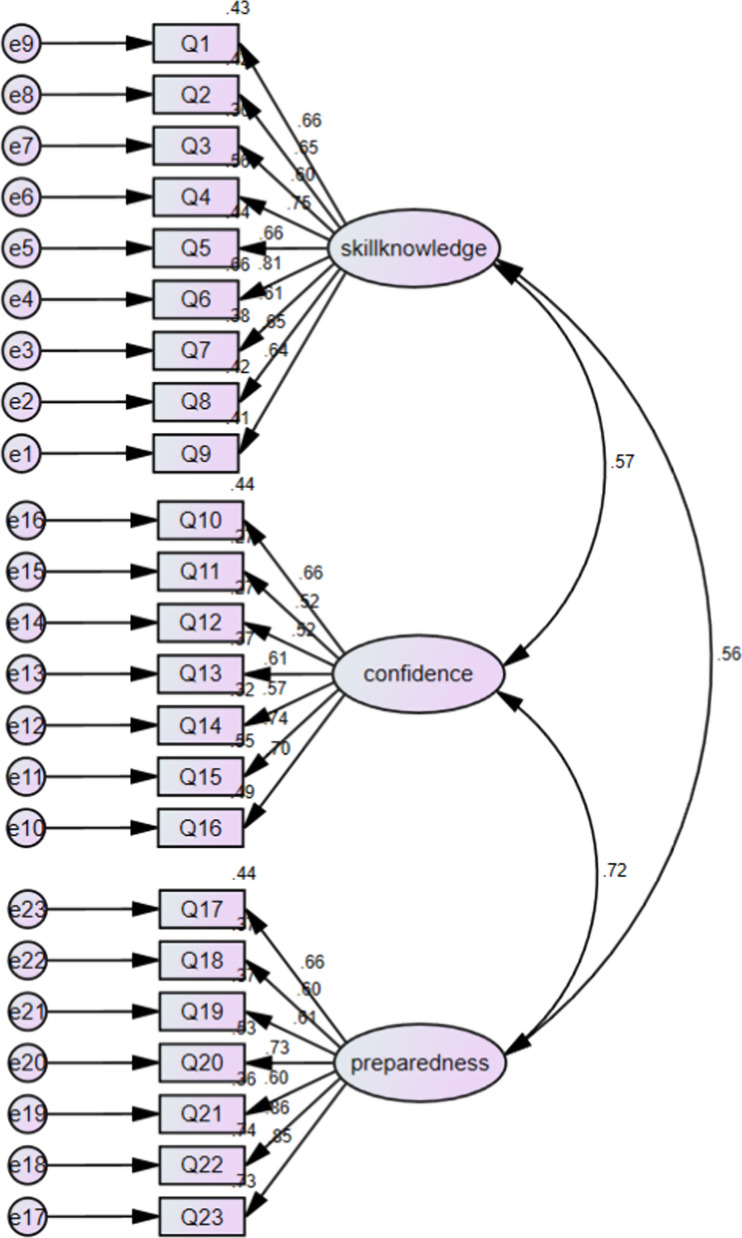
Confirmatory factor analysis model of the C-SCPI

**Fig. 2:**
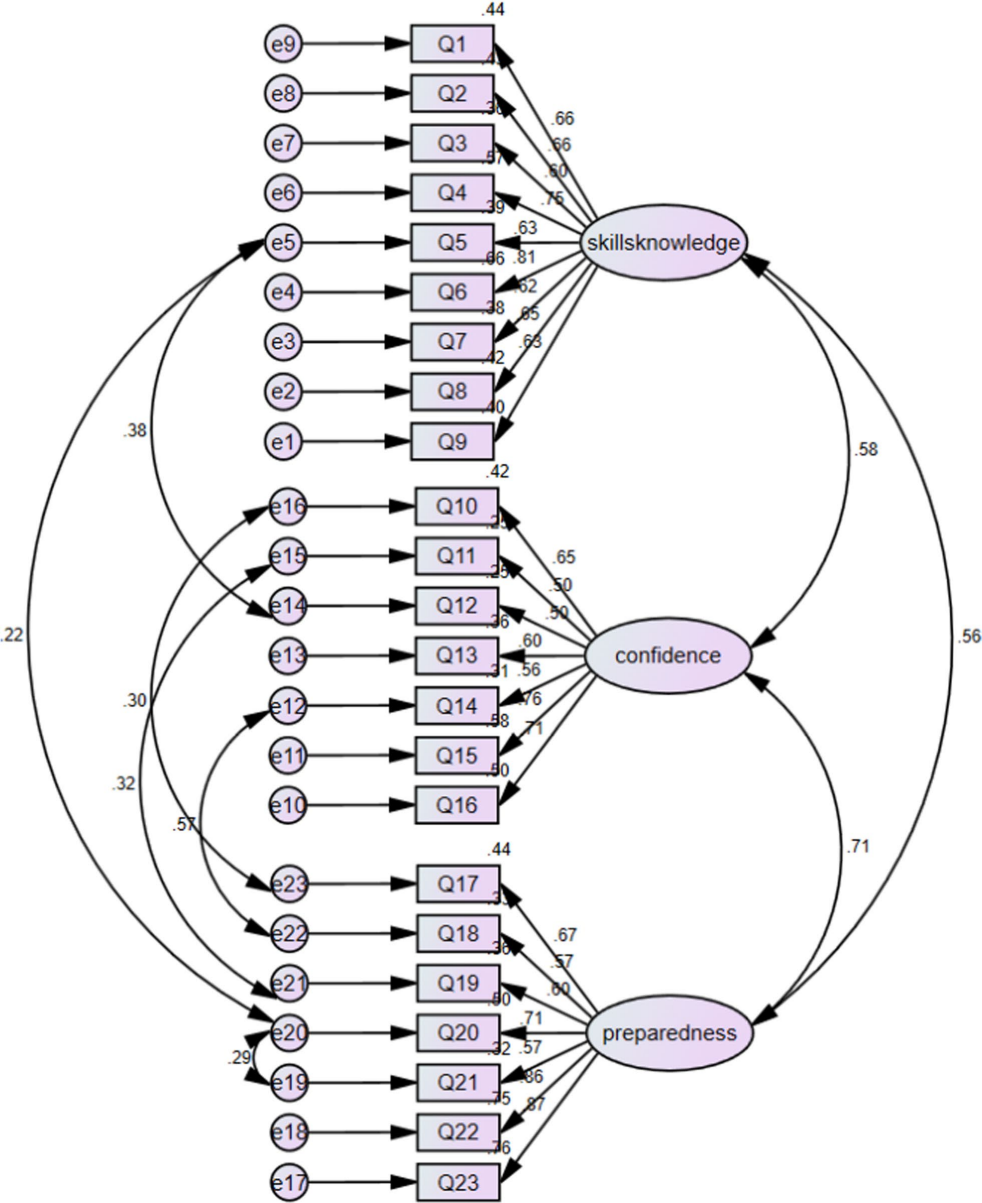
Confirmatory factor analysis model of the C-SCPI(modified model)

In the process of trans-language validation, no items were deleted. And based on the conceptual framework developed by the author of the original scale, CFA was performed to identify the underlying factor structure of the Chinese version of SCPI. All factor loadings based on a three-factor model of the 23 items were higher than the general standard 0.4. Initially, the model’s goodness of fit was unacceptable: *χ*^2^/df = 4.050, RMSEA = 0.090, CFI = 0.829, GFI = 0.820, TLI = 0.809, IFI = 0.830 (Fig. 1). The modification indices indicated that further improvements were possible by including more covariance parameters. On the basis of the original model and variable content, when the modification indices are higher than 4, it needs to be corrected to increase the path between the residuals to reduce the chi square value. Therefore, six covariance correlations were added to the pre-set model, and each fitting index was in line with its own acceptable reference: *χ*^2^/df = 2.775, RMSEA = 0.069, CFI = 0.903, GFI = 0.873, TLI = 0.889, IFI = 0.904 (Fig. 2).

### Content validity

In this study, six experts were invited to evaluate the correlation between items and their dimensions, and between items and self-management. The S-CVI value is 0.83. Except for item 21, the I-CVI values of all the other items are greater than 0.78, and 19 items have an I- CVI value of 1. The results indicate that the Chinese version of SCPI has good content validity and could better reflect the evaluation of self-management of diabetic patients. For item 21, we further modified it according to the experts’ opinions.

### Criterion validity and discriminative validity

There are positive correlations between the knowledge and skill part of SCPI and the total score of DKT in diabetic patients without insulin (r = 0.284, *p* < 0.001), and the total score of DKT in diabetic patients using insulin (r = 0.351, *p* < 0.001). The confidence part of SCPI has a good correlation with DES-SF (r = 0.376, *p* < 0.001). A positive correlation between the preparedness of SCPI and the total score of SDSCA (r = 0.465, *p* = 0.025) was also observed (Table 2).

**Table 2:** The reliability and criterion validity of Chinese version of SCPI

	Measure	Coefficient
Internal consistency for subscales		
Skills (9 items)	Cronbach’s alpha	0.88
Confidence (7 items)	Cronbach’s alpha	0.81
Preparedness (7 items)	Cronbach’s alpha	0.86
Overall (23 items)	Cronbach’s alpha	0.92
Test–retest reliability (n = 23)	Pearson’s correlation; *p* value	r = 0.61; *p* = 0.002
Criterion validity		
DES-SF (n = 242)	Spearman’s rank correlation; *p* value	0.376; *p* < 0.001
DKT^a^ (n = 121)	Spearman’s rank correlation; *p* value	0.284; *p* < 0.001
DKT^b^ (n = 118)	Spearman’s rank correlation; *p* value	0.351; *p* < 0.001
SDSCA (n = 23)	Spearman’s rank correlation; *p* value	0.465; *p* = 0.025

DKT a means diabetic patients without insulin completed the first 14 questions; DKT b means diabetic patients using insulin completed all 23 questions

HbA1c was divided into better control group (HbA1c ≤ 7) and poor control group (HbA1c > 7). Compared with the patients with poor blood glucose control, the patients with better control had higher scores of overall self-management and self-confidence (*p* < 0.05). There was no statistically significant difference found in scores of knowledge skills and self-management of behavior preparedness between the two groups (Table 3).

**Table 3:** Comparison of SCPI scores between patients with HbA1c ≤ 7.0% and HbA1c > 7.0%

	HbA_1C_ ≤ 7.0% (n = 79)	HbA_1c_ > 7.0% (n = 221)
Skills	5.3 ± 1.0	5.1 ± 1.0
Confidence	5.7 ± 0.6	5.5 ± 0.8*
Preparedness	6.2 ± 0.8	6.1 ± 0.8
Total	5.7 ± 0.7	5.5 ± 0.8*

*p < 0.05 compared to the patients who had with HbA1C ≤ 7.0%

### Internal consistency, test retest reliability and interpretability of the Chinese version of SCPI

The internal consistency of the Chinese version of SCPI is good with a Cronbach’s α of 0.92 for the total scale, and 0.81–0.88 for the each scale. Test–retest reliability conducted in 23 participants after 2 weeks is acceptable (r = 0.61; *p* = 0.002). No floor effects (> 15% of patients with a score of 1) or ceiling effects (> 15% of patients with a score of 7) were observed for the total score and the knowledge & confidence subscale scores, the ceiling effects of the preparedness subscales is 21%. The MCID values for the SCPI were 0.37 (0.5 SD), 0.21(SEM) using Cronbach’s α value.

## Discussion

Self-management is crucial for glycemic control in the diabetic patients. The SCPI focuses not only on the knowledge and self-confidence of self-management of diabetes mellitus, but also on the preparedness of individuals with diabetes to make a behavior change. In clinical practice, the scale reflects the self-management status of patients with diabetes quickly and provides the clues for healthy provider to formulate health education programs for people with diabetes.

In the phase one, the study presented the creation of a Chinese version of the SCPI (C-SCPI), which was translated and adapted from the original instrument through a systematic and rigorous process. We utilized the AAOS guideline [29] for cultural adaptation to Chinese. In the process of translation, although one of the translators has a B.A. in nursing, the other has completed postgraduate studies in English without a background in clinical care, which may eliminate any bias of current healthcare teaching and may better reflect the language used by the general population. There were some differences in the translations between the two translators, which might be due to the difference between their interpretations of natural expressions.

In the process of trans-language validation, experts’ guidance on the content of the scale is indispensable. They were asked to modify or provide appropriate wording when necessary [38]. In addition to language modification like change “how my diabetes medications (pills, injectables and/or insulin) work in my body” to “how diabetes medications (pills, injectables and/or insulin) reduce blood sugar in my body” to make semantic expressions clearer. At the same time, they also gave professional opinions on insulin dosage adjustment in light of the clinical practice in China. We also consulted the source scale authors about the autonomy of Canadian diabetic patients in insulin dose adjustment. The patients usually have specific education on dose adjustment when initiation insulin treatment. The patients can adjust their insulin dose on their own. The case is different in China clinical practice. We modified the expression of the item as a reminder to patients that they should adjust the insulin under the doctor’s advice.

The purpose of the interview was to use cognitive theory to understand how the respondents comprehend and answer the questionnaire items, find out the potential problems, and correct them [39]. The patients were able to provide us with practical insights. Most of the items of SCPI were well comprehended by the patients, but we also identified several items that were not well understood by the participants during the interview. The patients could not understand some professional words like “carbohydrates”, because they rarely hear “carbohydrates” in their daily life. Thus, we interpreted them to make them experiential equivalent. During the interview, some patients thought that the content of diabetes self-management was only “eating less and exercising more”, while ignoring medication and daily blood glucose monitoring. So we have adjusted some expressions to reduce patients’ doubts and the vagueness of diabetes management in order to adapt the hospital settings in China and to be understood easily. Feedback from patients is crucial and may led to linguistic changes that improve the acceptability of the final scales.

The results of criterion validity demonstrated the self-confidence dimension of C-SCPI correlated well with the DES-SF, which has been extensively used as an empowerment in diabetes instrument throughout the world, which is similar to the results of Mbuagbaw [24], and indicates that patients with higher levels of empowerment have higher confidence in self-management of diabetes. The results showed that there was a positive correlation between the behavioral preparation part of SCPI and the total score of SDSCA (r = 0.465, *p* = 0.025), indicating that the predictive validity of the preparedness part of SCPI was good, which could better reflect the behavioral preparation of patients in the next month, so the medical staff would provide more accurate health education.

Tools for measuring self-care behavior in diabetic patients should be able to distinguish between patients with good and poor blood glucose control. The study shows that SCPI could effectively distinguish the self-management behavior of patients with different blood glucose control outcomes. In terms of patients’ self-confidence level, it is consistent with previous relevant research results [40, 41], which indicate that the patients with better glycation control and self-management level have higher empowerment ability and self-management confidence in disease. In addition, 47.7% of the 300 patients with glycosylation records in this study were diabetic patients aged over 60 years. In the 143 patients over 60 years old, 65% of them had poor blood glucose control. Another study has showed that the level of diabetes knowledge is negatively with age [42]. Elderly should be the focus of health education. There is no statistically significant difference between the two groups in the preparedness part of SCPI, which may be related to the potential change of glycosylated hemoglobin in the future. Moreover, the subjects of this survey were all inpatients, and the patients who have received hospitalization treatment and health education may have higher scores for the preparedness in the next month after discharge. In general, the C-SCPI is a reliable tool to evaluate self-management level of diabetic patients, and it also suggests that helping diabetic patients improve their self-management level will improve the outcome of blood glucose control.

The model could be specified to be even more theoretically consistent by allowing more pathways between the items [43]. The study added six covariance coefficients to the preset model, which may be related to the same potential latent. Knowledge, attitude and practice of diabetes self-management may be related. Although knowledge and skills, self-confidence and behavior preparedness belong to three different dimensions in this scale, the factors of the three dimensions may be potentially correlated. For example, Q14 and Q18 measures patients’ daily physical exercise with Q14 focusing on patients’ confidence in exercise and Q18 on patients’ preparation for exercise (see Fig. 2). If a patient has the confidence to exercise, he may put exercise into action next month, so there may be a potential correlation between Q14 and Q18. Similarly, Q5 and Q12, Q5 and Q20 measure hypoglycemia prevention; Q11 and Q19 measure the patient’s regulation of stress; Q10 and Q17 focus on the diet of patients. Taking into account the theoretical underpinnings of the SCPI, the statistical significance of all the items in the model, and the fitting index has been greatly improved after the data has been modified, the C-SCPI with three factor structure is acceptable. The internal consistency of the C-SCPI is satisfying with a Cronbach’s α of 0.92 for the total scales [44]. This finding corresponds well with those reported in the original English version [45]. Interpretability measures the capacity of a questionnaire to be interpreted from quantitative scores or change in scores to a qualitative meaning. MCID value is a minimum change score at or above which the change can be considered (by some definition) to be important [46, 47]. When the change value of SCPI score exceeded MCID, the self-management ability of diabetes mellitus changed. The ceiling effects of the preparedness subscales is 21% may be due to the awareness of the serious harm of diabetes during hospitalization, which indicates that these patients are well prepared for behavior change.

The test–retest reliability coefficient of the scale is just above the non-acceptable level may be due to the knowledge & skill dimension. After completing the scale for the first time, the patients may consulted the unclear knowledge points with professionals, then they had their own thinking and understanding of diabetes and mastered the relevant knowledge of diabetes. The test–retest reliability may be unstable because of the results of the first measurement, further investigation is needed to strictly verify test–retest reliability of the SCPI. It also showed that SCPI has an educational effect on the self-management of diabetic patients.

There are some limitations to the current assessment that should be acknowledged. The original SCPI scale was developed and validated in both type 1 and type 2 diabetic patients. However, only type 2 diabetic patients were investigated in the current study. The application of the scale in type 1 diabetic patients in China needs further study and discussion. Because the samples in the study mainly came from the inpatients of a university-affiliated hospital, the applicability of the samples in the outpatient and community diabetes patients needs further investigated in the future. The number of patients who had test and retest and patients who completed SDSCA in this study is quite small, which needs to be further verified in future studies. Nevertheless, the SCPI can be further applied in health education projects to test the impact of the scale on improving blood glucose level and self-management behavior of patients.

In the future, the MCID values of each dimension can be further calculated and verified by the Anchor-based approaches. And we expect SCPI can be used in the “cloud platform” to improve the self-management and monitoring system of diabetic patients in the future.

## Conclusion

Our study followed the strict guidelines of cross-cultural adaption of the scale. After the initial version of the scale was formed, the reliability and validity of the SCPI scale were verified. The C-SCPI has good internal consistency and satisfied criterion validity and discriminative validity. It provides an effective measurement tool and theoretical basis for the investigation of self-management level and behavioral preparation of diabetic patients.

## Supplementary information

**Additional file 1**. Process of cross-cultural adaptation & General information of experts & Modified content after interviewing.

## Data Availability

Data are presented in the manuscript and Additional file 1.

## Abbreviations

SCPI: The LMC Skills, Confidence & Preparedness Index
AAOS: American Academy of Orthopaedic Surgeons Evidence Based Medicine Committee
IDF: The International Diabetes Federation
DCP: Diabetes Care Profile
DKT: Diabetes Knowledge Test
DES: Diabetes Empowerment Scale
AADE: The American Diabetes Education Association
CPG: Clinical Practice Guidelines
SET: Self-efficacy theory
HbA1c: Glycosylated Haemoglobin
CFA: Confirmatory factor analysis
GFI: Goodness-of-Fit Index
CFI: Comparative Fit Index
IFI: Incremental fit index
TLI: Tucker-Lewis index
RMSEA: Root mean square error of approximation
MCID: Minimal clinically important difference

## References

[1] International Diabetes Federation. IDF Diabetes Atlas, 9th edn. Brussels, Belgium: 2019. http://www.diabetesatlas.org.

[2] Wang L, Gao P, Zhang M, Huang Z, Zhang D, Deng Q (2017). Prevalence and Ethnic pattern of diabetes and prediabetes in China in 2013. JAMA.

[3] Washington G, Wang-Letzkus MF (2009). Self-care practices, health beliefs, and attitudes of older diabetic Chinese Americans. J Health Hum Serv Adm.

[4] Hewitt J, Smeeth L, Chaturvedi N, Bulpitt CJ, Fletcher AE (2011). Self management and patient understanding of diabetes in the older person. Diabet Med.

[5] Carpenter R, DiChiacchio T, Barker K (2018). Interventions for self-management of type 2 diabetes: an integrative review. Int J Nurs Sci.

[6] Ayele K, Tesfa B, Abebe L, Tilahun T, Girma E (2012). Self-care behavior among patients with diabetes in Harari, Eastern Ethiopia: the health belief model perspective. PLoS ONE.

[7] Ji JJ, Liu L, Lou QQ, Yuan XD, Yao P, Zhang DY (2014). Self-management behaviors and glycemic control in patients with type 2 diabetes mellitus. Chin J Nurs.

[8] Wu JL, Cheng KY, Lyu WB (2017). The research progress on the current situation of self -blood glucose monitoring at home and abroad and its influencing factors. Nurs J Chin PLA.

[9] Qin MY, Liang YR, Gong X (2019). Research on diabetes management and influencing factors among middle-aged and the elderly diabetes patient. Chin Prim Health Care.

[10] Group of Diabetes Education and Management, Diabetes Society of Chinese Medical Association (2017). Chinese consensus on self-administered prescriptions for type 2 diabetes. Chin J Diabetes Mellitus.

[11] Lou QQ, Yang LL, Shao AX (2005). Behavior change and diabetes mellitus. Chin J Diabetes Mellitus.

[12] Fitzgerald JT, Funnell MM, Hess GE, Barr PA, Anderson RM, Hiss RG (1998). The reliability and validity of a brief diabetes knowledge test. Diabetes Care.

[13] Garcia AA, Villagomez ET, Brown SA, Kouzekanani K, Hanis CL (2001). The Starr County Diabetes Education Study: development of the Spanish-language diabetes knowledge questionnaire. Diabetes Care.

[14] Dunn SM, Bryson JM, Hoskins PL, Alford JB, Handelsman DJ, Turtle JR (1984). Development of the diabetes knowledge (DKN) scales: forms DKNA, DKNB, and DKNC. Diabetes Care.

[15] Polonsky WH, Anderson BJ, Lohrer PA, Welch G, Jacobson AM, Aponte JE (1995). Assessment of diabetes-related distress. Diabetes Care.

[16] Van Der Ven NC, Weinger K, Yi J, Pouwer F, Adèr H, Van Der Ploeg HM (2003). The confidence in diabetes self-care scale: psychometric properties of a new measure of diabetes-specific self-efficacy in Dutch and US patients with type 1 diabetes. Diabetes Care.

[17] Anderson RM, Fitzgetald JM, Gruppen LD, Funnell MM, Oh MS (2003). The diabetes empowerment scale-short form (DES-SF). Diabetes Care.

[18] Toobert DJ, Hampson SE, Glasgow RE (2000). The summary of diabetes self-care activities measure: results from 7 studies and a revised scale. Diabetes Care.

[19] Charron-Prochownik D, Zgibor JC, Peyrot M, Peeples M, McWilliams J, Koshinsky J (2007). The Diabetes Self-management Assessment Report Tool (D-SMART): process evaluation and patient satisfaction. Diabetes Educ.

[20] Schmitt A, Gahr A, Hermanns N, Kulzer B, Huber J, Haak T (2013). The Diabetes Self-Management Questionnaire (DSMQ): development and evaluation of an instrument to assess diabetes self-care activities associated with glycaemic control. Health Qual Life Outcomes.

[21] Fitzgerald JT, Davis WK, Connell CM, Hess GE, Funnell MM, Hiss RG (1996). Development and validation of the Diabetes Care Profile. Eval Health Prof.

[22] Wang WJ, Liu XX, Chen B, Li CF, Feng NP (2016). Development on the diabetes self-management knowledge, attitude, and behavior assessment scale (DSKAB). Chin J Prev Med.

[23] Mayberry R, Willock RJ, Boone L, Lopez P, Qin H, Nicewander D (2010). A high level of patient activation is observed but unrelated to glycemic control among adults with type 2 diabetes. Diabetes Spectr.

[24] Mbuagbaw L, Aronson R, Walker A, Brown RE, Orzech N (2017). The LMC Skills, Confidence & Preparedness Index (SCPI): development and evaluation of a novel tool for assessing self-management in patients with diabetes. Health Qual Life Outcomes.

[25] Aronson R, Li A, Brown RE (2019). Optimizing diabetes self-management using the novel skills, confidence, and preparedness index (SCPI). Diabetes Care.

[26] Cheng AY, Canadian Diabetes Association Clinical Practice Guidelines Expert Committee (2013). Canadian Diabetes Association 2013 clinical practice guidelines for the prevention and management of diabetes in Canada. Can J Diabetes.

[27] Bandura A (1977). Self-efficacy: towards a unifying theory of behavior change. Psychol Rev.

[28] Yin B (2007). Cross-theoretical model of health behavior change. Chin Ment Health J.

[29] Beaton DE, Bombardier C, Guillemin F, Ferraz MB (2000). Guidelines for the process of cross-cultural adaptation of self-report measures. Spine J.

[30] Chinese Diabetes Society (2018). Guidelines for the prevention and control of type 2 diabetes in China (2017 Edition). Chin J Pract Internal Med.

[31] Sousa VD, Rojjanasrirat W (2011). Translation, adaptation and validation of instruments or scales for use in cross-cultural health care research: a clear and user-friendly guideline. J Eval Clin Pract.

[32] Chen AL, Zhang ZL, Liao ZH, Wan LH, Deng WP, Yuan YH (2006). Self-management and quality of life in patients with diabetes mellitus. Chin J Behav Med Sci.

[33] Hu BB, Lou QQ, Tian Y, Zhang QW, Zhu JY (2011). Study on empowerment and its influencing factors among diabetes inpatients. Chin J Nurs.

[34] Hua L, Zhu WP (2014). Verification of the reliability and validity of chinese version of diabetes self-management activities questionnaire. Nurs J Chin PLA.

[35] Brown TA (2015). Confirmatory factor analysis for applied research.

[36] Polit DF, Beck CT (2006). The content validity index: are you sure you know what's being reported? Critique and recommendations. Res Nurs Health.

[37] Kulthanan K, Chularojanamontri L, Tuchinda P, Rujitharanawong C, Maurer M, & Weller K. Validity, reliability and interpretability of the Thai version of the urticaria control test (UCT). Health and quality of life outcomes.2016;14:61. https://doi.org/10.1186/s12955-016-0466-y. 10.1186/s12955-016-0466-y PMC4831163 27075142

[38] Sigurdardottir AK, Benediktsson R (2008). Reliability and validity of the Icelandic version of the Problem Area in Diabetes (PAID) Scale. Int J Nurs Stud.

[39] Jobe JB (2003). Cognitive psychology and self-reports: models and methods. Qual Life Res.

[40] Strychar I, Elisha B, Schmitz N (2012). Type 2 diabetes self-management: role of diet self-efficacy. Can J Diabetes.

[41] Zou YX, Deng AH, Zhong Y, Sun Y, Luo HC (2013). The impact of Empowerment Ability on Self-management Behavior in Type 2 Diabetes. Chin Nurs Manag.

[42] Guo HJ, Wang Q, Mao T, Zhu J, Xu XP, Wang B et al. The cognitive status and influence factors of diabetes mellitus with different populations. Chin J Health Educ. 2018; 34(08):694–698+708. 10.16168/j.cnki.issn.1002-9982.2018.08.005.

[43] Kwok C, Fethney J, White K (2012). Confirmatory factor analysis of the Chinese Breast Cancer screening beliefs questionnaire. Cancer Nurs.

[44] Lance CE, Butts MM, Michels LC (2006). The sources of four commonly reported cutoff criteria: what did they really say?. Org Res Methods.

[45] Aronson R, Brown RE, Jiandani D, Walker A, Orzech N, Mbuagbaw L (2018). Assessment of self-management in patients with diabetes using the novel LMC Skills, Confidence and Preparedness Index (SCPI). Diabetes Res Clin Pract.

[46] Sloan JA, Symonds T, Vargas-Chanes D, Friendly B (2003). Practical guidelines for assessing the clinical significance of health related quality of life changes within clinical trials. Ther Innov Regul Sci.

[47] Beaton DE, Boers M, Wells GA (2002). Many faces of the minimal clinically important difference (MCID): a literature review and directions for future research. Curr Opin Rheumatol.

