# Supplementary material for: Reliability and validity of the Chinese version of the LMC Skills, Confidence & Preparedness Index (SCPI) in patients with type 2 diabetes
Source: Health Qual Life Outcomes. 2021 Jan 20;19:25. doi: 10.1186/s12955-020-01664-x (PMC7816447; doi:10.1186/s12955-020-01664-x)
Supplement: Supplementary file 1 — Additional file 1. Process of cross-cultural adaptation & General information of experts & Modified content after interviewing. [file 12955_2020_1664_MOESM1_ESM.docx]

**Process of cross-cultural adaptation**


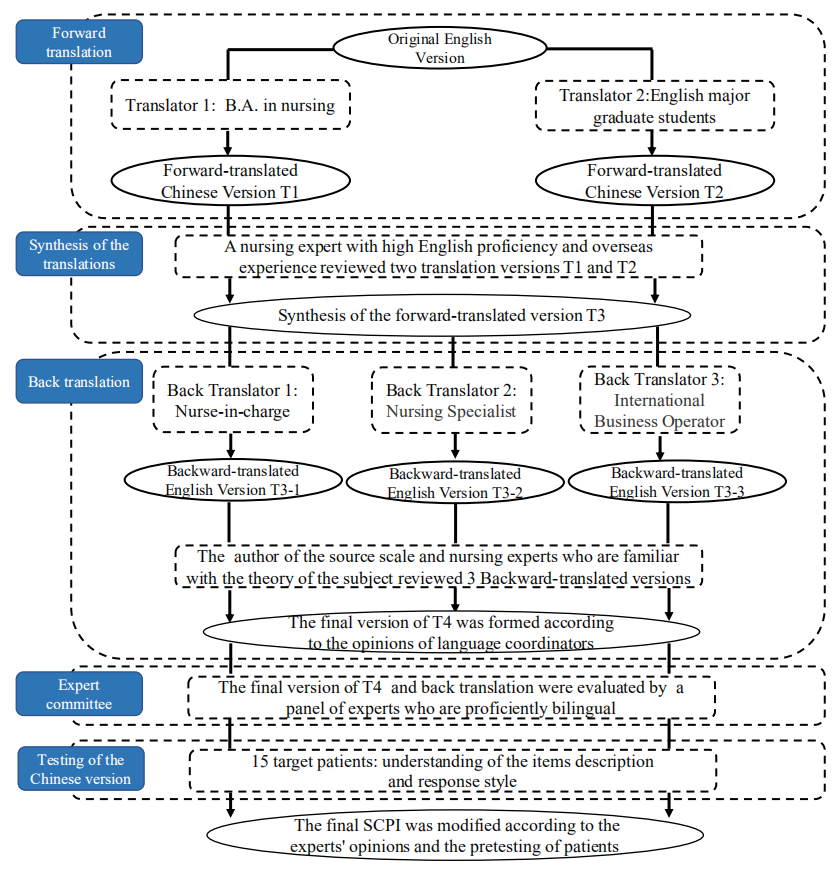


**General information of experts**

|  | Educational background | Positional titles | Professional background | Years of professional work |
| --- | --- | --- | --- | --- |
| 1 | Ph.D. and M.D | Chief physician | Endocrinology | 17 |
| 2 | Ph.D. | Associate Professor | Nursing education and research | 19 |
| 3 | M.D. | Deputy Chief physician | Endocrinology | 26 |
| 4 | M.D. | Chief physician | Prevention and control of chronic diseases | 28 |
| 5 | MSN | Co-chief nurse | Endocrinology | 14 |
| 6 | MSN | Co-chief nurse | Clinical care | 16 |

**Modified content after interviewing**

| Original entry | Translated entry | Modified entry | Reasons for changes |
| --- | --- | --- | --- |
| 1.I know how to plan meals that have the best balance between carbohydrates, proteins and vegetables.  6.When I am sick, I know what to do differently with my diabetes management.  7.I know how stress can affect my diabetes management.  8.When I look at my blood sugars over a given week, I can explain what my blood sugar pattern is.  16.When something out of my normal routine happens, I am confident that I can problem-solve and keep my diabetes on track. | 我知道如何安排能最好地平衡碳水化合物、蛋白质和蔬菜的饮食  当我生病时，我知道如何有区别地应对我的糖尿病管理  我知道压力是如何影响到我的糖尿病管理的  当我看到我某一周的血糖记录时我能解释我的血糖变化模式(或规律)  当日常常规活动被打破时，我能自信地解决问题并将糖尿病维持在正常轨道。 | 我知道如何制订饮食计划，更好地平衡碳水化合物（含淀粉/糖的食物）、蛋白质和蔬菜。  当我身体不适时，我知道如何有区别地应对我的糖尿病管理（如饮食、用药、运动和血糖监测）。  我知道各种压力是如何影响到我的糖尿病管理（如饮食、用药、运动和血糖监测）的。  当我看到我某一周的血糖记录时我能解释我的血糖变化情况（比如血糖过高或过低的原因）。  当生活规律被打破时，我能自信地解决问题并将血糖控制在目标范围。 | Old people could not understand “carbohydrate”  Some patients were not sure about the scope of self-management of diabetes. How to define the scope of diabetes management was simply to control blood sugar or comprehensive management.  The meaning of stress is not clear, and the scope of diabetes management is not clear.  Patients could not understand “what my blood sugar pattern is”，What does the pattern mean?  Some patients raised that “keep my diabetes on track” was not in line with Chinese language habits. |
| 19.Within the next month, I will make a list of stress management options that work for me. | 在下个月，我将制定一系列对我有用的压力管理办法。 | 在下个月，我将找到一些适用于自己的缓解压力的办法。 | Older people did not understand “stress management” |
